# An Interesting Rare Case Report of Primary Amelanotic Melanoma With Distant Metastasis

**DOI:** 10.7759/cureus.72803

**Published:** 2024-10-31

**Authors:** Kenneth Singh, Athira Gopinathan, Sivamarieswaran R

**Affiliations:** 1 General Surgery, SRM Medical College Hospital and Research Centre, Chennai, IND

**Keywords:** diagnosis, immunotherapy, prognosis, rectal malignant melanoma, surgical resection, treatment

## Abstract

Anorectal malignant melanoma (ARMM) is a rare and highly aggressive type of melanoma that originates in the anorectal area. It represents a small fraction of all melanoma cases and is often associated with poor prognosis due to its late presentation and challenging treatment options. Rectal malignant melanoma typically presents with symptoms such as rectal bleeding, pain, and obstructive symptoms. Diagnostic imaging and histopathological examination are crucial for accurate diagnosis, often requiring a combination of endoscopy, biopsy, and immunohistochemical staining. Treatment primarily involves surgical resection, though adjuvant therapies such as chemotherapy, immunotherapy, and radiation may be utilized depending on disease stage and metastasis. The prognosis remains guarded, with survival rates significantly lower compared to cutaneous melanoma, attributed to late-stage diagnosis and high likelihood of metastatic spread. Here we are reporting a case of ARMM presenting late with lung, skeletal, and liver metastasis.

## Introduction

Anorectal malignant melanoma (ARMM) is an exceptionally rare and highly aggressive melanoma type. Cancerous growths known as melanomas can arise in any area of the body that contains melanocytes, including the skin, eyes, nasal cavity, oropharynx, vagina, urinary tract, rectum, and anus [1,2]. Unlike the more common cutaneous melanomas that arise from skin cells, ARMM develops from melanocytes in the rectal mucosa. This contributes to significant challenges in diagnosis and management, as the condition often eludes early detection and is frequently diagnosed at an advanced stage. ARMM comprises less than 1% of all rectal malignancies and an even smaller proportion of melanoma cases overall [3]. It is more commonly diagnosed in middle-aged to elderly individuals, with no clear gender predominance. The risk factors associated with rectal malignant melanoma are not as well-defined as those for cutaneous melanoma, but some studies suggest that it may share in common with other types of melanomas, including genetic predispositions and certain environmental factors.

The clinical presentation of rectal malignant melanoma often includes symptoms like pain, rectal bleeding, as well as changes in bowel habits, which can be mistaken for more common benign rectal disorders. The non-specific nature of these symptoms frequently leads to delay in diagnosis [4]. Patients may present with localized disease or with metastases to regional lymph nodes or distant organs, complicating treatment and affecting prognosis.

Accurate diagnosis demands a high degree of suspicion as well as a thorough diagnostic methodology, including endoscopic examination and biopsy. Histopathological analysis, supported by immunohistochemical staining, is essential to differentiate rectal malignant melanoma from other rectal tumors and to confirm the diagnosis. Imaging tests including computed tomography (CT) and magnetic resonance imaging (MRI) scans are also utilized to evaluate the degree and extent of the disease and to assist treatment planning because of the aggressive nature of the disease [5].

The cornerstone of treatment for rectal malignant melanoma management is still surgical resection. However, due to the potential for late diagnosis and metastasis, adjuvant therapies, including chemotherapy, immunotherapy, and radiation, are often considered based on individual patient factors and disease stage. The prognosis for rectal malignant melanoma is generally poor compared to cutaneous melanoma, largely due to its late presentation and propensity for metastasis [2]. Here we are reporting a case of ARMM presenting late with skeletal, lung, and liver metastasis.

## Case presentation

**Figure 1:**
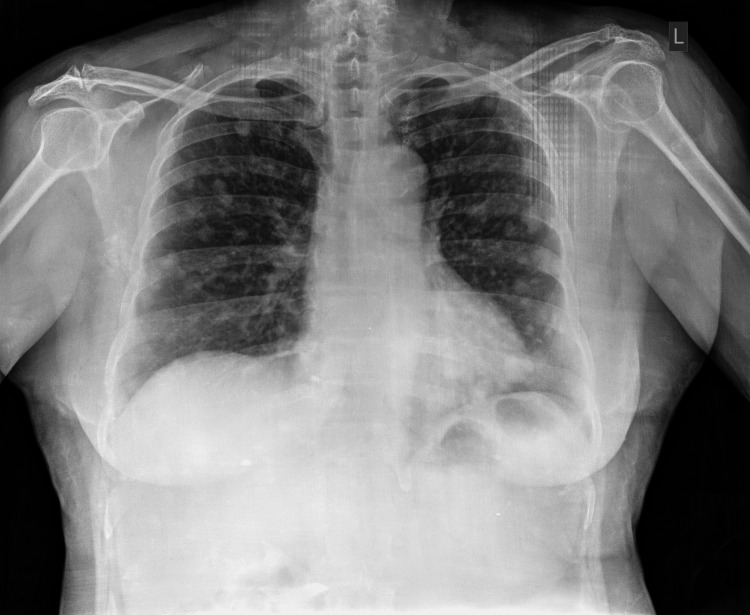
X-ray chest showing multiple coin-like lesions diffusely scattered in bilateral lung fields suggestive of metastasis

A female patient of 60-year-old presented with symptoms of rectal bleeding and intermittent peri-anal pain that had been occurring for the past six months. These symptoms were previously assessed by another healthcare provider and incorrectly labeled as a case of anal fissure. She also had vague abdominal pain and occasional loose stools. On per rectal examination, an ulcerated nodular mass felt 2 cm above the anal verge that bled on touch with increased sphincter tone. On examination of the abdomen, no mass or organomegaly was felt. The liver function test was deranged with mildly elevated total bilirubin and liver enzymes. X-ray of the chest (Figure 1) showed multiple coin-like lesions diffusely scattered in bilateral lung fields suggestive of metastasis.

**Figure 2:**
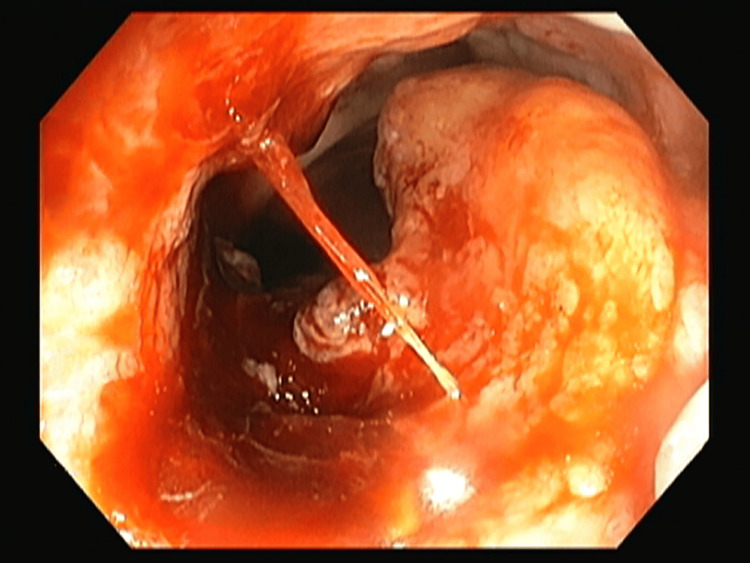
Colonoscopy picture showing mass at 7'o clock position 2 cm above the anal verge

**Figure 3:**
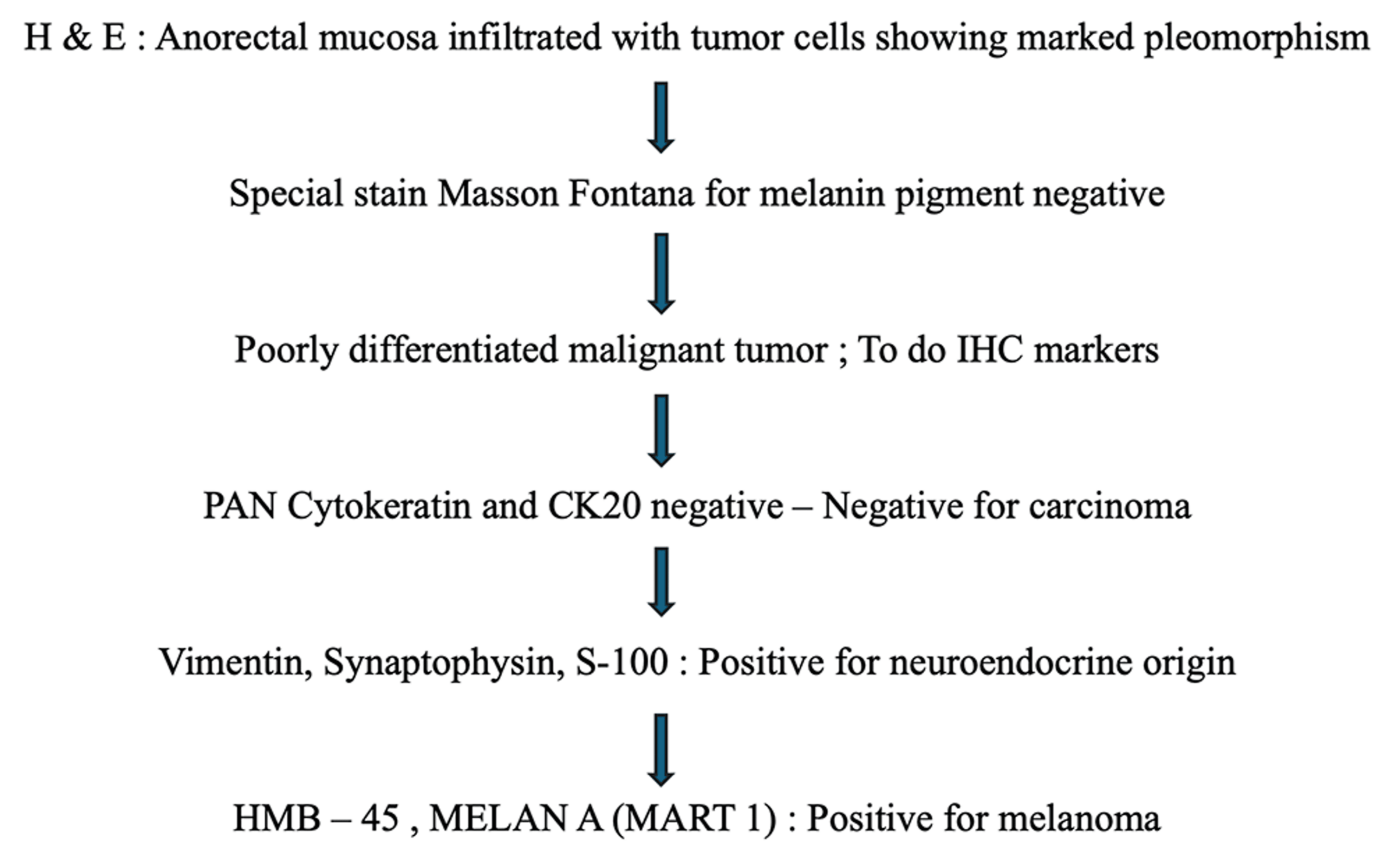
Tissue diagnosis of colonoscopy biopsy H&E: hematoxylin and eosin; CK: cytokeratin

**Figure 4:**
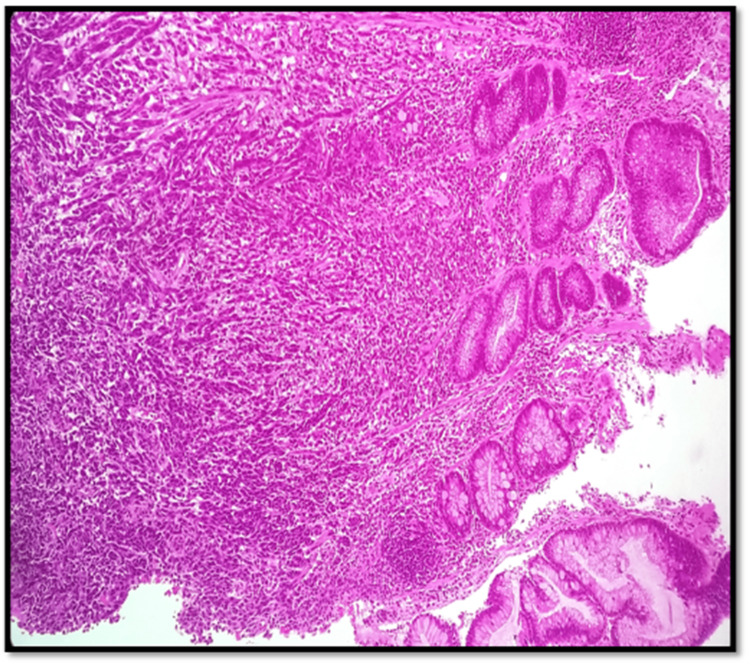
H&E 10× slide examination shows anorectal mucosa infiltrated by tumor cells arranged in cords, trabeculae, and in sheets H&E: hematoxylin and eosin

**Figure 5:**
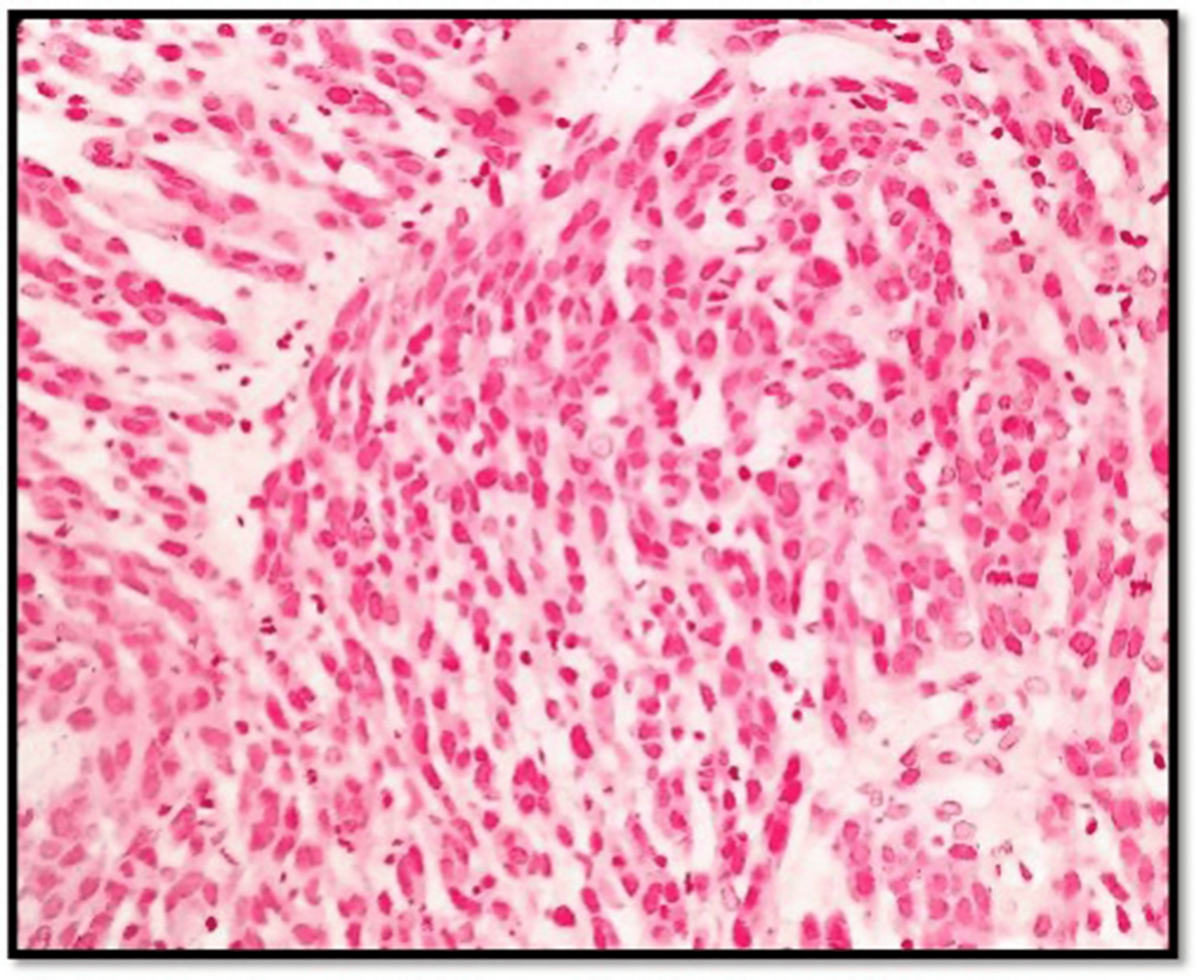
Negative image of special Masson Fontana stain for melanin pigment

**Figure 6:**
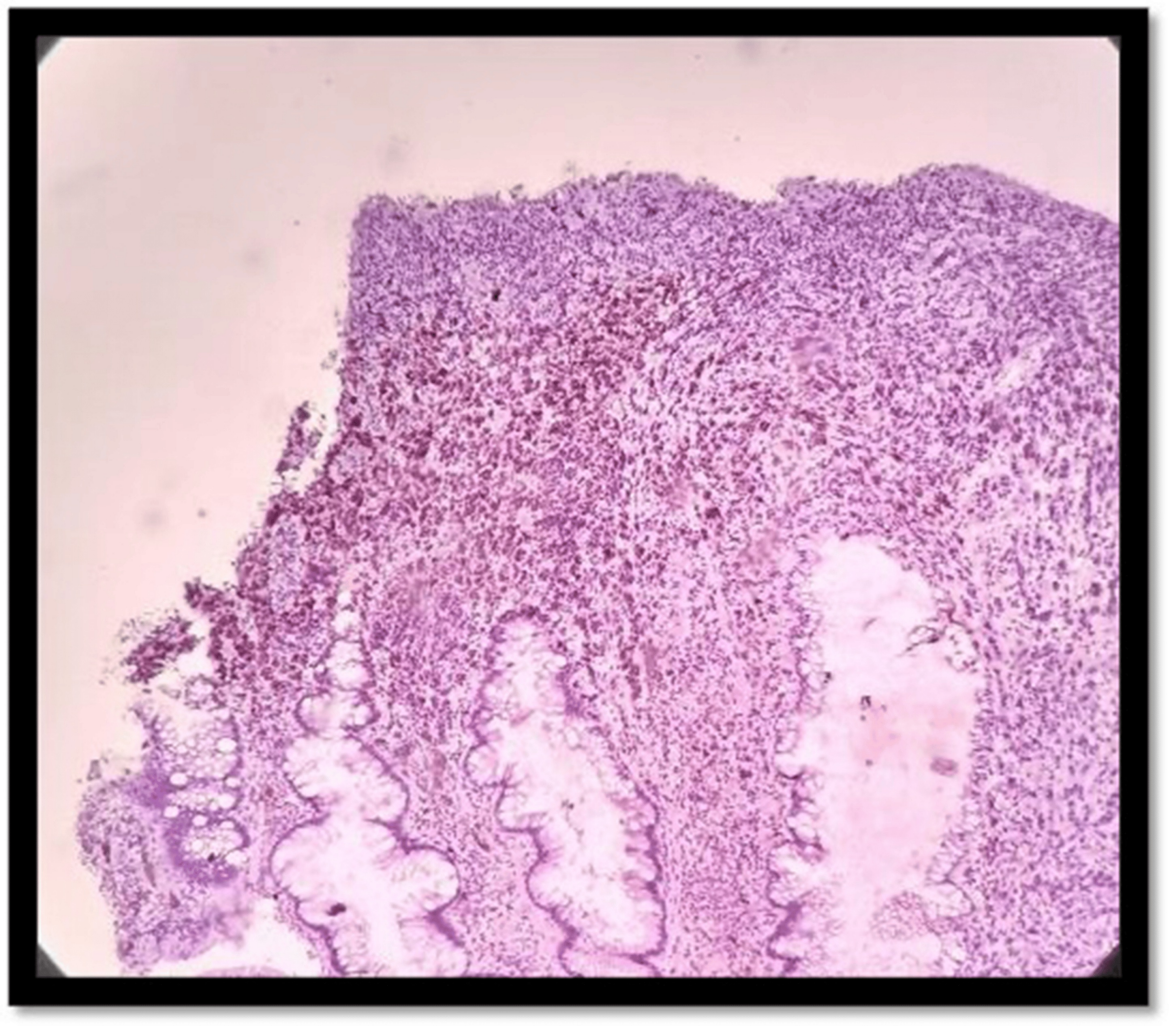
HMB-45 positive in tumor cells suggestive of melanoma HMB-45: human melanoma black 45

**Figure 7:**
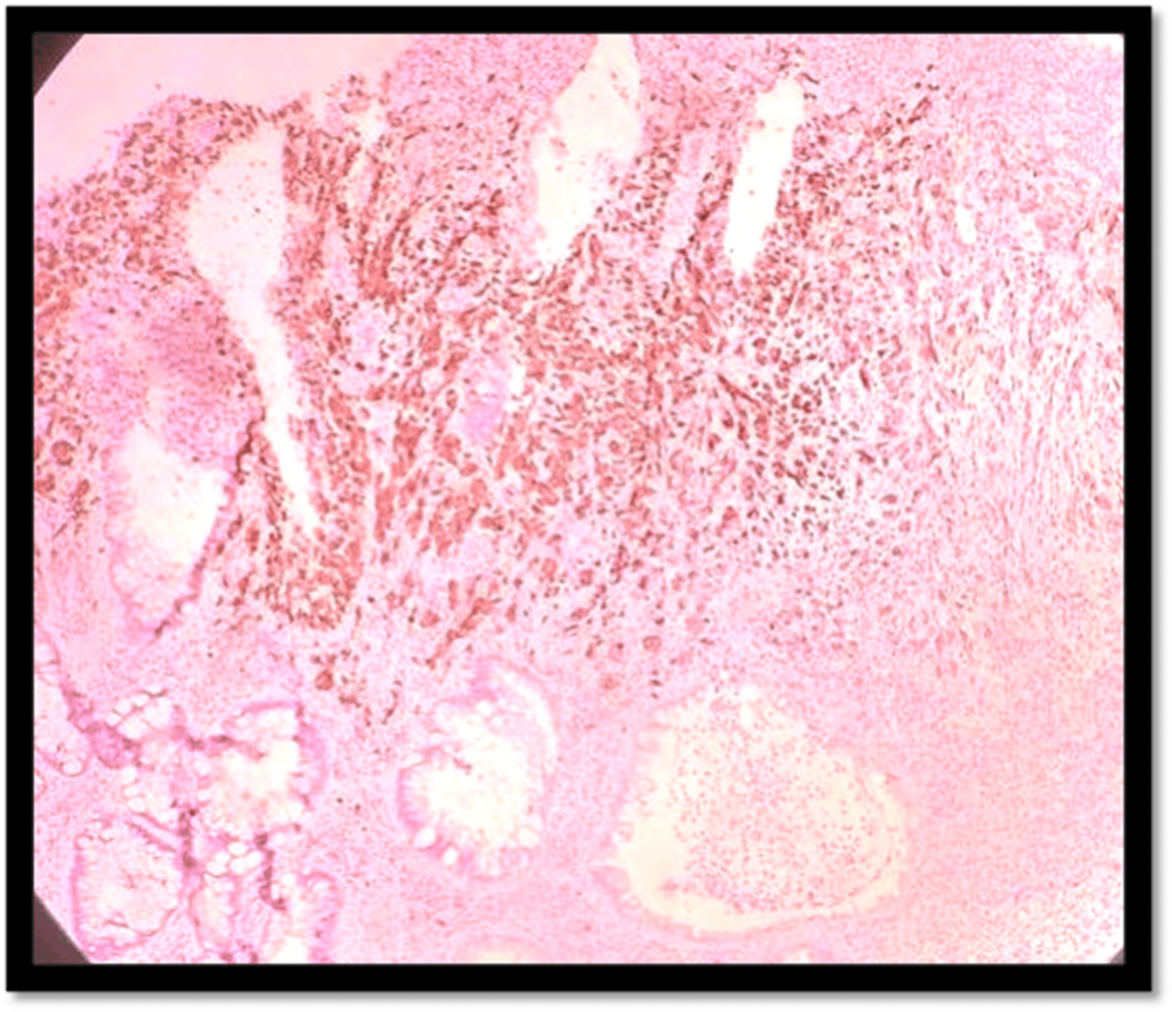
MELAN-A (MART-1) seen in tumor cells, which is a melanocyte-specific cytoplasmic protein MELAN-A (MART 1): Melanoma-associated Antigen Recognized by T cells

**Figure 8:**
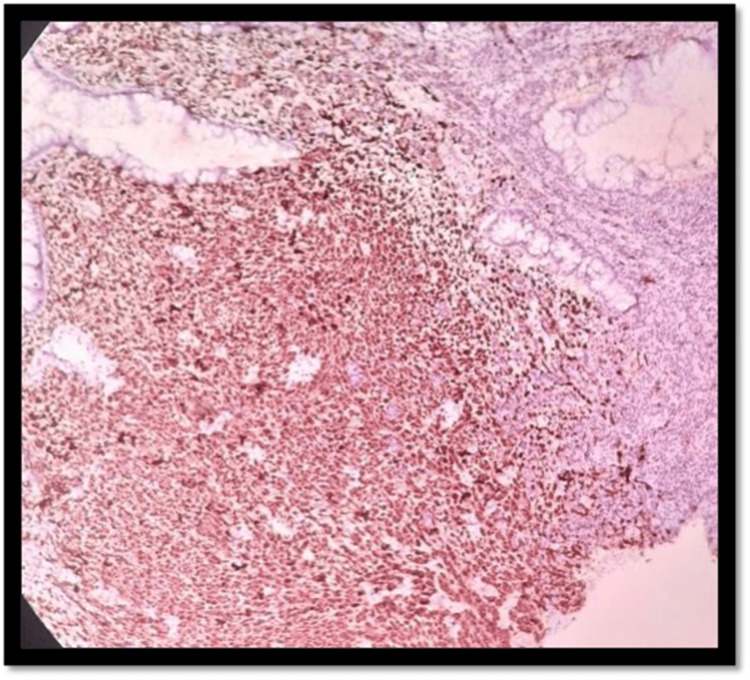
S-100 positive in tumor cells suggestive of neuroendocrine origin and melanoma

**Figure 9:**
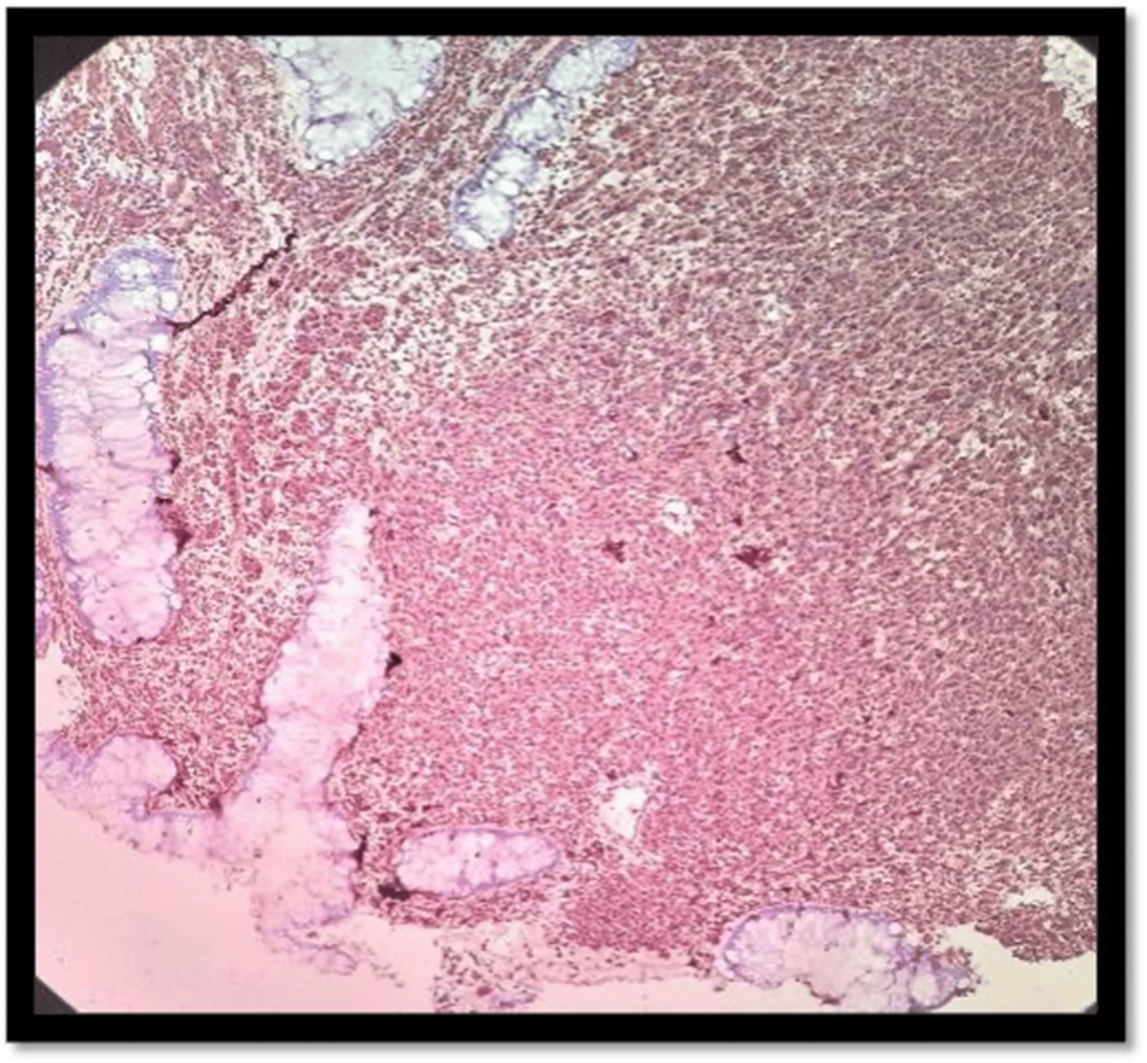
Vimentin positive in tumor cells also suggestive of mesenchymal origin

**Figure 10:**
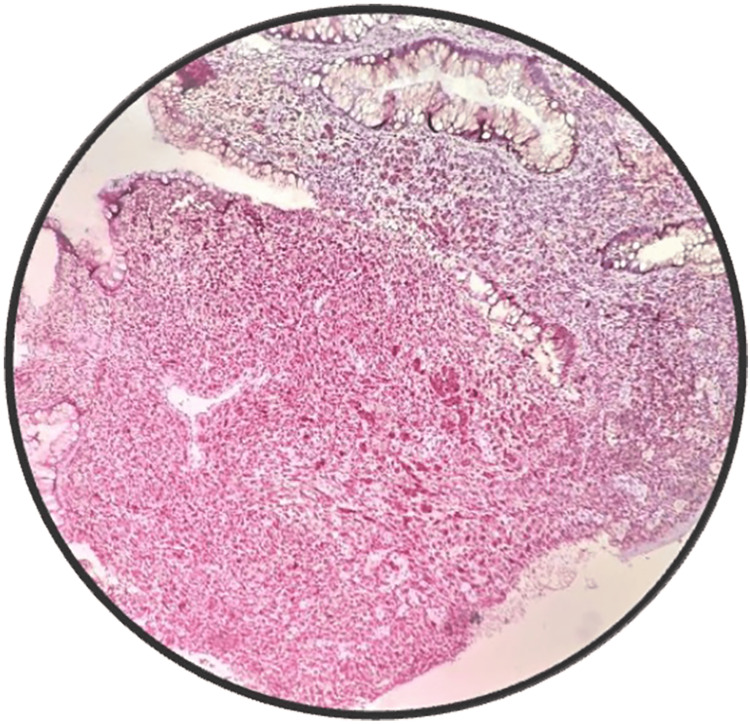
Synaptophysin positive in tumor cells suggestive of neuroendocrine origin

**Figure 11:**
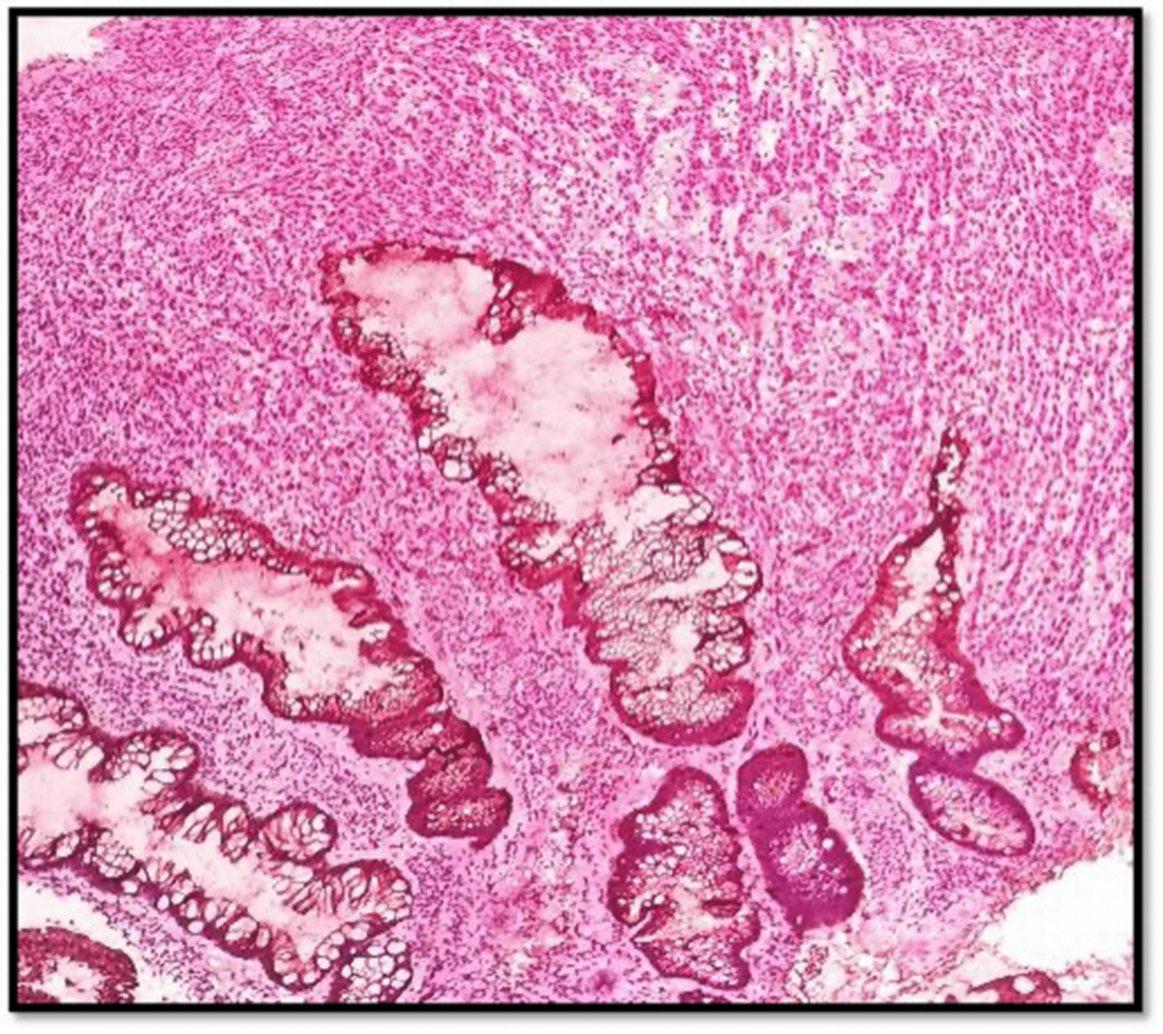
Pan-CK negative in tumor cells ruling out carcinoma CK: cytokeratin

**Figure 12:**
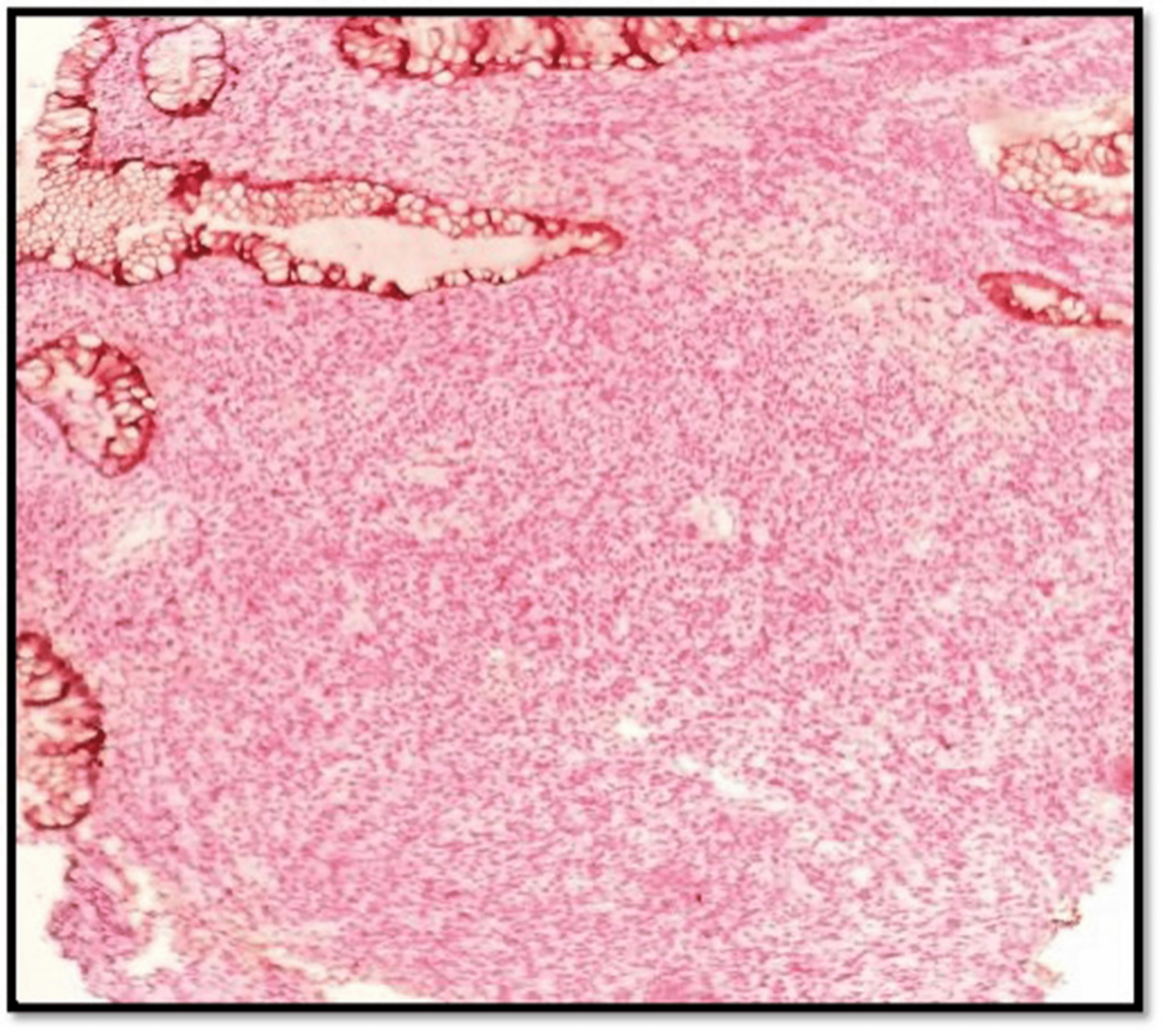
CK-20 negative in tumor cells ruling out carcinoma CK: cytokeratin

The contrast-enhanced CT of the abdomen and chest showed Iliac and obturator nodal metastasis noted. Bilateral lung metastasis was noted. Colonoscopy revealed an ulcerated nodular growth at 7 o’clock position 2 cm above the anal verge shown in Figure 2. Microscopic findings with its inference on adding different stains to tissue are shown in Table 1. A schematic representation of the biopsy examination is shown in Figure 3. Histopathology examination revealed a poorly differentiated malignant tumor after staining with hematoxylin & eosin (H&E) in Figure 4, and a special stain Mason Fontana in Figure 5. Immunohistochemistry (IHC) markers were done and showed positivity for (human melanoma black 45) HMB-45 in Figure 6; Melanoma-associated Antigen Recognized by T cells (MELAN-A/MART-1) in Figure 7; S-100 in Figure 8; and vimentin in Figure 9. Synaptophysin was in Figure 10 and was negative for pan-cytokeratin (CK) in Figure 11. CD31 in Figure 12, which ruled out the potential for vascular lesions and adenocarcinoma was finally diagnosed as malignant melanoma (amelanotic type) with neuroendocrine differentiation. Hence, the disease can be staged as stage 3 according to the Ballantyne scoring system.

**Table 1:** Microscopic findings on using different stains with their inference H&E: hematoxylin and eosin; CK: cytokeratin; MELAN-A (MART 1): Melanoma-associated Antigen Recognized by T cells; HMB-45: human melanoma black-45

Name of stain	Finding	Inference
H&E	Positive	Poorly differentiated malignant tumor cells
Special Mason Fontana	Negative	Melanin not seen
Pan-CK	Negative	Rules out carcinoma
CK 20	Negative	Rules out carcinoma
Vimentin	Positive	Mesenchymal origin
Synaptophysin	Positive	Neuroendocrine differentiation
S-100	Positive	Marker of melanocytes and Schwann cells
HMB-45	Positive	Marker of melanoma
MELAN-A (MART 1)	Positive	Melanocyte specific

The PET-CT findings confirmed the malignant nature of the tumor, indicating widespread metastasis involving the mesorectal, superior rectal, left inguinal, and right obturator lymph nodes, as well as distant metastasis to the liver, lungs, and lumbar vertebrae. Our patient underwent palliative chemotherapy but succumbed to her illness within two months of initiation of chemotherapy.

## Discussion

Anorectal melanoma (ARM) was first documented in 1857. Gastrointestinal tract malignant melanoma is an exceptionally uncommon occurrence, with 50% of cases specifically affecting the anorectal region. ARM exhibits highly aggressive activity and is associated with a very poor prognosis due to delayed diagnosis [3]. Often resulting in a fatal conclusion, as shown in our patient. 

Melanomas are more frequently found in the rectum than in the anus, and they tend to progress rapidly in the rectal region. Melanoma originates from melanocytes, which move away from the neural crest or mucocutaneous junction to the skin during embryonic development. A potential reason for the presence of this cancer in this specific area of the body could be the absence of a specialized area for stem cells in the bulge region of the melanomas seen in the glabrous skin [6]. Melanoma accounts for between 0.5% and 4% of all malignancies in this area. After the skin and retina, this site is the third most common main site for melanoma. An ARM is more frequent in females and often occurs in the fifth to sixth decade of life. An ARM can be hard to identify grossly and may be treated incorrectly as hemorrhoid, rectal polyp, or occasionally mass following protrusion through the anal orifice with ulceration, similar to our patient who was previously misdiagnosed as having a fissure in ano. Most lesions are pigmented and polypoidal in nature, but a histopathologic study of a hemorrhoidectomy specimen is necessary to identify those that are amelanotic. Because amelanotic melanoma lacks pigmentation, it can be mistakenly diagnosed as a sarcoma or carcinoma, while being a distinct form of malignant melanoma. HMB-45 immunohistochemical staining has been shown to be helpful in the histological as well as cytological diagnosis of amelanotic melanoma, according to recent reports [1]. As a result, amelanotic malignant melanomas could have villous carcinoma-like characteristics [6]. Biopsy is essential to a proper diagnosis in these situations. Immunohistochemical tests play a crucial role in the process of diagnosis. Positive expression of protein S-100, melanoma antigen HMB-45, and vimentin is commonly observed in malignant melanoma. The possibility of adenocarcinoma, on the other hand, is indicated by positive staining for cytokeratin, epithelial membrane antigen, and carcinoembryonic antigen [6]. In our patient, an accurate diagnosis was made due to the presence of IHC markers such as HMB-45, MELAN-A, and S-100, which indicate melanoma; synaptophysin and vimentin, which indicate neuroendocrine origin; and pan-CK and CK20, which ruled out carcinoma.

Upon diagnosis, it is seen that approximately 70% of patients with ARM have metastases, most likely because the anus contains a large number of vascular and lymphatic systems. When it comes to squamous cell carcinoma of the anus, mesorectal lymph nodes are more commonly impacted than inguinal lymph nodes [7]. Metastasis was observed in both the mesorectal and inguinal nodes of our patient. Distant metastases commonly involve the liver, lungs, brain, and bones. The presence of BRAF mutation was not identified in anal mucosal melanomas, but it was observed in 33% of cutaneous melanomas [8]. MRI is a diagnostic method of choice for assessing the extent of anal melanomas tumor invasion. 

The most effective treatments for anal melanomas are abdominoperineal resection (APR) and wide local excision (WLE), which are the two most commonly performed surgical procedures [9]. Historically, APR was carried out. Another minimally invasive alternative is endoscopic mucosal resection (EMR), which is limited to the removal of tumors located in the mucosal and superficial submucosal layers. This procedure is particularly suitable for elderly patients who are frail and unable to undergo the more aggressive WLE or APR. EMR can lead to long-term survival in cases of small tumors that have not deeply invaded surrounding tissue. WLE has, meanwhile, also been utilized recently for localized cancers where negative tumor margins can be obtained [2]. Because hidden systemic dissemination is nearly always present, this more cautious strategy is justified by the desire to protect the anal sphincter [4,6]. WLE is less invasive, requires no stoma formation, and has a faster rate of recovery with minimal impact on bowel, urine, and sexual function [7]. The best course of action is difficult to identify due to the condition's rarity and lack of randomized controlled trials, which has generated controversy [7].

The utilization of adjuvant therapy in combination with local excision resulted in a decrease in mortality. Studies have reported that a combination of chemotherapy or radiotherapy with surgery has shown benefits for patients. Interferon, interleukin-2 (IL-2), dacarbazine, paclitaxel, and temozolomide have demonstrated favorable efficacy in treating anal melanoma [9]. The combination of IL-2 and ipilimumab, administered at a dosage of 3 mg/kg, demonstrated efficacy in patients with unresectable anal melanomas. There is a limitation of information regarding the use of further systemic treatment in individuals with mucosal melanoma. There is an absence of randomized trials specifically studying the usage of ipilimumab for mucosal melanoma [10]. Palliative surgical procedures, such as local segmental resection or diversion colostomy, can be carried out [9].

Prognostic factors involve tumor necrosis, tumor size greater than 20 mm, and tumor thickness (Breslow greater than 2 mm) are associated with a higher chance of disease recurrence and indicate a more aggressive tumor [7]. Additional features associated with a worse prognosis include tumor perineural invasion, inguinal lymph node presence, persistence of symptoms longer than three months, and histologically confirmed amelanotic melanoma. Tumor thickness of less than 4 mm is related to improved disease-free and overall survival, whereas tumor thickness of 4 mm or above is associated with a lower disease-free survival rate and a higher risk of systemic recurrence, according to a study of patients with long-term survival [6]. The literature that is currently accessible suggests that India may have a higher incidence than the West [10].

## Conclusions

ARMM is an uncommon, aggressive cancer that presents challenging treatment and diagnostic decisions. Our patient had non-specific symptoms and presented late with distant metastasis. Early detection remains critical for improving outcomes, necessitating heightened awareness among clinicians. Ongoing research into more effective treatment modalities and early diagnostic techniques is essential to enhance survival rates and quality of life in affected patients. The prognosis remains poor even if diagnosed early.
